# Effects of sudden walking perturbations on neuromuscular reflex activity and three-dimensional motion of the trunk in healthy controls and back pain symptomatic subjects

**DOI:** 10.1371/journal.pone.0174034

**Published:** 2017-03-20

**Authors:** Juliane Mueller, Tilman Engel, Steffen Mueller, Josefine Stoll, Heiner Baur, Frank Mayer

**Affiliations:** 1 University Outpatient Clinic, Sports Medicine & Sports Orthopaedics, University of Potsdam, Germany; 2 Bern University of Applied Sciences, Health, Physiotherapy, Bern, Switzerland; Semmelweis Egyetem, HUNGARY

## Abstract

**Background:**

Back pain patients (BPP) show delayed muscle onset, increased co-contractions, and variability as response to quasi-static sudden trunk loading in comparison to healthy controls (H). However, it is unclear whether these results can validly be transferred to suddenly applied walking perturbations, an automated but more functional and complex movement pattern. There is an evident need to develop research-based strategies for the rehabilitation of back pain. Therefore, the investigation of differences in trunk stability between H and BPP in functional movements is of primary interest in order to define suitable intervention regimes. The purpose of this study was to analyse neuromuscular reflex activity as well as three-dimensional trunk kinematics between H and BPP during walking perturbations.

**Methods:**

Eighty H (31m/49f;29±9yrs;174±10cm;71±13kg) and 14 BPP (6m/8f;30±8yrs;171±10cm;67±14kg) walked (1m/s) on a split-belt treadmill while 15 right-sided perturbations (belt decelerating, 40m/s^2^, 50ms duration; 200ms after heel contact) were randomly applied. Trunk muscle activity was assessed using a 12-lead EMG set-up. Trunk kinematics were measured using a 3-segment-model consisting of 12 markers (upper thoracic (UTA), lower thoracic (LTA), lumbar area (LA)). EMG-RMS ([%],0-200ms after perturbation) was calculated and normalized to the RMS of unperturbed gait. Latency (T_ON_;ms) and time to maximum activity (T_MAX_;ms) were analysed. Total motion amplitude (ROM;[°]) and mean angle (A_mean_;[°]) for extension-flexion, lateral flexion and rotation were calculated (whole stride cycle; 0-200ms after perturbation) for each of the three segments during unperturbed and perturbed gait. For ROM only, perturbed was normalized to unperturbed step [%] for the whole stride as well as the 200ms after perturbation. Data were analysed descriptively followed by a student´s t-test to account for group differences. Co-contraction was analyzed between ventral and dorsal muscles (V:R) as well as side right:side left ratio (S_right_:S_left_). The coefficient of variation (CV;%) was calculated (EMG-RMS;ROM) to evaluate variability between the 15 perturbations for all groups. With respect to unequal distribution of participants to groups, an additional matched-group analysis was conducted. Fourteen healthy controls out of group H were sex-, age- and anthropometrically matched (group H_matched_) to the BPP.

**Results:**

No group differences were observed for EMG-RMS or CV analysis (EMG/ROM) (p>0.025). Co-contraction analysis revealed no differences for V:R and S_rigth_:S_left_ between the groups (p>0.025). BPP showed an increased T_ON_ and T_MAX_, being significant for Mm. rectus abdominus (p = 0.019) and erector spinae T9/L3 (p = 0.005/p = 0.015). ROM analysis over the unperturbed stride cycle revealed no differences between groups (p>0.025). Normalization of perturbed to unperturbed step lead to significant differences for the lumbar segment (LA) in lateral flexion with BPP showing higher normalized ROM compared to H_matched_ (p = 0.02). BPP showed a significant higher flexed posture (UTA (p = 0.02); LTA (p = 0.004)) during normal walking (A_mean_). Trunk posture (A_mean_) during perturbation showed higher trunk extension values in LTA segments for H/H_matched_ compared to BPP (p = 0.003). Matched group (BPP vs. H_matched_) analysis did not show any systematic changes of all results between groups.

**Conclusion:**

BPP present impaired muscle response times and trunk posture, especially in the sagittal and transversal planes, compared to H. This could indicate reduced trunk stability and higher loading during gait perturbations.

## Background

Non-specific back pain (BP) is a major burden on health systems of western societies, with a lifetime prevalence of about 85% and frequently leading to disability in 10% to 15% of all patients affected [1–4]. In etiology, potential causes for back pain are discussed including repetitive micro trauma and insufficiency of the muscle-tendon complex based on inadequate postural and neuromuscular control, reduced maximum trunk strength capacity and trunk muscle fatigue during dynamic loading [5–7]. In addition, these factors are defined as important, contributing to the stability of the trunk [8–11]. Therefore, great emphasis has been placed on the importance of trunk stability, especially in situations requiring compensation of (unexpected) high loading induced e.g. by perturbations [8–11]. Stability provided by the trunk muscles is considered meaningful in counteracting sudden, unexpected loading during daily life as well as dynamic, high-intensity activities [8,12]. Hence, optimizing neuromuscular core stability is considered beneficial for protection against sudden, repetitive and excessive overloading of the trunk [8,9,12,13,14,15].

When compensating for sudden external (un-)expected perturbations, delayed muscle onset, increased co-contractions, and increased EMG variability has been shown in back pain patients (BPP) [9,16–18]. However, most of the studies applied the load directly to the trunk, mainly in non-dynamic situations (e.g. standing or sitting) [17,19]. Therefore, the transferability of these results to dynamic loading, daily life or sports situations applied by the lower limbs cannot be validated, and has to be discussed critically due to the quasi-static and limited functional load application. Sudden loading during gait, therefore, might be a more suitable situation in which to analyse differences between healthy controls (H) and back pain patients (BPP) [20–23]. The human gait is described as an automated and stable movement pattern (high intra-individual reproducibility) with more functional and complex demands on the neuromuscular system and kinematic chain of the trunk compared to the quick-release experiments. Moreover, there is an evident need to develop research-based strategies for the prevention and rehabilitation of back pain. Therefore, the investigation of differences in trunk function and stability between healthy and back pain patients in functional movements is of primary interest in order to define adequate intervention regimes.

The analysis of trunk kinematics and posture comparing patients and healthy participants has been discussed as beneficial for extracting the mechanical factors that may be associated with the development, persistence and recurrence of back pain [13,14,20]. However, inconsistent results regarding movement patterns and kinematic variability during gait have been found [24–27]. Vogt et al. [27] reported a higher stride-to-stride variability of all lumbar movement planes in lower back pain patients, while the absolute range of motion was unchanged compared to healthy controls. In addition, Steel et al. [26] reported a higher movement variability during gait in patients compared to healthy controls only in the sagittal and transverse planes. Moreover, some studies reported that symptomatic participants display reduced lumbar rotational movements [20], while others showed that lower BP increases spine or pelvis rotation [28].

In summary, it is unclear whether back pain patients (BPP) suffer from delayed muscle reflex response and higher trunk movement variability with sudden dynamic loading during gait. Consequently, the purpose of this study was to analyse the effects of sudden walking perturbations on neuromuscular reflex activity and three-dimensional motion of the trunk in healthy controls and back pain symptomatic subjects (BPP). It is hypothized that BPP have increased reflex response times to sudden loading while walking with increased neuromuscular activity, especially of the abdominal muscles. In addition, an increased range of motion of all segments with higher movement variability in the sagittal plane in BPP is expected. Besides, the neuromuscular and kinematic response pattern to a walking perturbation was analysed in comparison of H and BPP.

## Material and methods

### Participants

The investigation was conducted at the University Outpatient Clinic and participants were recruited from the Outpatient Clinic (e.g. students and/or academic workers from the university population receiving physical examination, recreational athletes receiving annual health check-ups), using flyers (displayed at the university cafeteria and sports facilities) and existing contacts with training groups at the Olympic Center. Therefore, enrolled participants were physically active and recreational trained athletes, 18 to 50 years of age, of both sexes. 97 participants were initially recruited for the study. After receiving an explanation of written informed consent, protocols and additional oral information from the study coordinator, 94 (37m / 57f; 29±9yrs; 173±10cm; 71±13kg) participants agreed to partake. All participants read and signed a written informed consent form before voluntary participation. The University Potsdam Ethical Commission approved the study.

In accordance with the grading score of the pain questionnaire, participants were assigned to the healthy controls (H; Korff grades 0 and 1) or back pain symptomatic subjects group (BPP; Korff grades 2-4) [29, 30, 31]. Therefore, 80 participants were allocated to the healthy control (H) and 14 to the back pain symptomatic subjects (BPP) group. Anthropometrics and pain sub scores (pain intensity/disability score) are presented in Table 1.

**Table 1:** Anthropometrics and back pain status of healthy controls (H;H_matched_) and back pain symptomatic subjects (BPP).

Group	N	Sex (f/m)	Age [yrs]	Body height [cm]	Body weight [kg]	Korff Pain Intensity Score	Korff Disability Score
H	80	49/31	29 ± 9	174 ± 10	71 ± 13	17 ± 13	8 ± 11
BPP	14	8/6	30 ± 8	171 ± 10	67 ± 14	50 ± 13	41 ± 18
H_matched_	14	8/6	28 ± 8	170 ± 8	67 ± 12	13 ± 12	8 ± 11

With respect to the unequal distribution of participants included in both groups, an additional matched-group analysis was conducted. Therefore, the equal number (N = 14) of healthy controls out of group H was sex-, age- and anthropometrically matched (group H_matched_) to the number of back pain symptomatic subjects (BPP; N = 14).

### Measurement protocol

**Fig 1:**
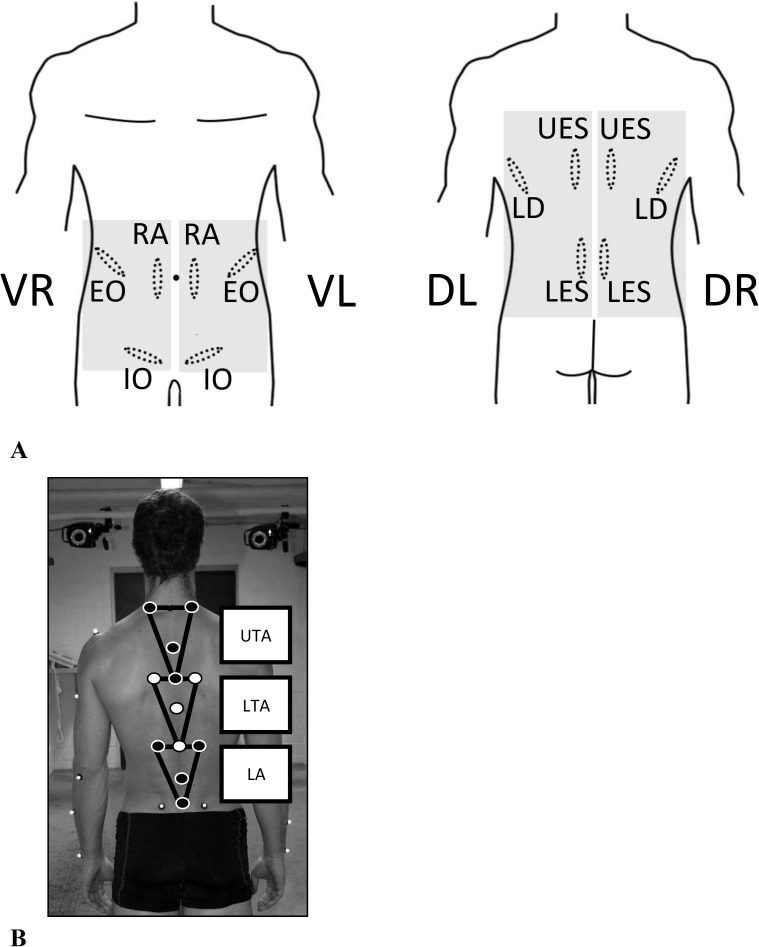
A. 12-lead EMG-trunk-setup. (Mm rec. abd. (RA), obl. ext. abd. (EO), obl. int. abd (IO) of left and right side) and 6 dorsal (Mm erec. spinae thoracic (T9; UES)/lumbar (L3; LES), latis. dorsi (LD) of left and right side; VR: RA, EO, IO of right side; VL: RA, EO, IO of left side; DR: UES, LES, LD of right side; DL: UES, LES, LD of left side). B. Kinematic trunk model (Müller et al. 2015).

After receiving an anthropometric assessment, all participants answered an online-based (ProWebDB, Germany) version of the graded chronic pain questionnaire (von Korff) valid to determine the presence of back pain [29,30,31]. The (back) pain questionnaire consisted of 7 items, including pain intensity and disability (recently and last 3 months) [29,30,31]. Six items are conducted of a numeric rating scale ranging from 0 (no pain/disability) to 10 (highest pain/ disability (incapable of doing anything)). Sub scores of pain and disability are calculated. Furthermore, participants can get classified into one of the five hierarchical pain and disability grades ranging from low pain/disability (grade 0) to high pain/disability scores (grade IV) [29,30,31]. This was followed by a clinical examination conducted by an experienced physician to ensure eligibility for the upcoming stumbling protocol. Participants were then prepared for EMG and kinematic analysis of the trunk. EMG electrodes were positioned over twelve trunk muscles (Fig 1B; see EMG analysis section). Twelve reflective markers were precisely positioned over bony structures (Fig 1A; see kinematic analysis section) [25]. Subject preparation was followed by a standardized walking perturbations protocol beginning with a warm-up and familiarization procedure where the participants walked 5 minutes at 1m/s on a split belt treadmill (Woodway, Weil am Rhein, Germany) without perturbation [25]. Next, each subject walked for about 10 minutes at a baseline velocity of 1m/s; while walking, 15 right- and left-sided perturbations were randomly applied 200ms after initial heel contact triggered by a plantar pressure insole (Pedar X, Novel, Munich, D). This ensures that participants are perturbed in the early phase of the gait cycle (weight acceptance) and single support phase bearing already full load of body weight on the foot. During perturbation, one of the treadmill belts decelerated to a velocity of -1m/s (amplitude: 2 m/s) resulting in a deceleration of -40m/s^2^ for 50ms, returning to baseline velocity after an additional 50ms. Detailed information of the perturbation characteristic are detailed elsewhere [25]. For the data analysis, only right-sided perturbations were analysed due to direct triggering of the perturbations by the plantar pressure insole used only in the right shoe. Left-sided perturbations were also applied to ensure that participants did not adapt their normal walking pattern to only right-sided perturbations.

Overall, participants were commanded to walk as natural as possible on the treadmill while randomly perturbations will be applied. As a consequence, participants walked on the treadmill while knowing that perturbations will be applied but not knowing when (time), where (leg) and how (treadmill belt movement direction). In addition, participants were instructed to compensate the stimuli, avoid falling and aiming to get back to normal upright walking pattern within the following three to four steps [32]. No further instructions on arm, leg or trunk movements were given. For safety reasons, all participants worn a waist belt connected to an emergency stop release.

### EMG analysis

Trunk muscle activity was assessed with a 12-lead surface EMG [17,33]. The setup included 6 ventral (Mm rec. abd. (RA), obl. ext. abd. (EO), obl. int. abd (IO) of left and right side) and 6 dorsal (Mm erec. spinae thoracic (T9; UES)/lumbar (L3; LES), latis. dorsi (LD) of left and right side) muscles. Muscular activity was analyzed using bilateral and bipolar surface EMG (bandpass filter: 5 – 500 Hz; sampling frequency: 4000 Hz, amplification: overall gain: 1000; myon, Switzerland). Before electrodes (AMBU Medicotest, Denmark, Type N-00-S, inter- electrode distance: 2 cm) were applied, the skin was shaved, slightly exfoliated to remove surface epithelial layers, and finally disinfected. In addition, skin resistance was controlled by measuring skin impedance (<5 kΩ). The longitudinal axes of the electrodes were in line with the presumed direction of the underlying muscle fibers. The signal was rectified before calculation of the amplitudes. No additional filter was applied post processing.

The root mean square analysis as well as the calculation of the onset of muscular activity served as primary outcomes for EMG analysis.

**Fig 2:**
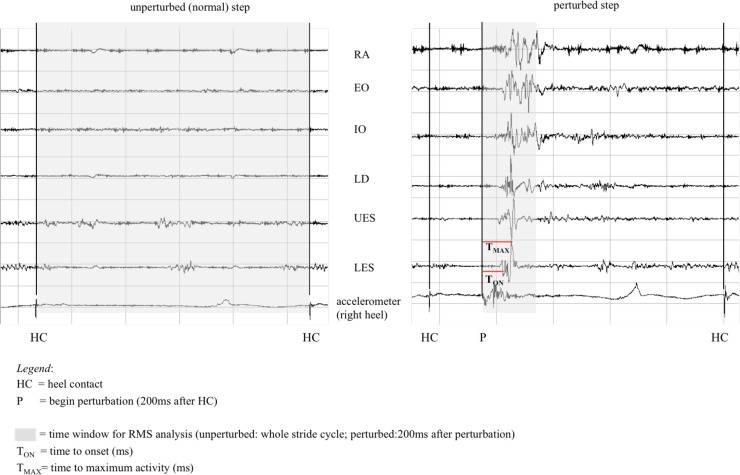
Exemplarily EMG signal for the 6 right-sided trunk muscles (raw signal of 1 perturbation for one subject) including visualization of EMG outcome measures (EMG-RMS, T_ON_, T_MAX_).

The mean amplitude for each muscle was calculated out of the first 5 unperturbed strides and the 15 perturbed strides of the walking perturbations protocol. The root mean square (RMS; [%]) within the first 200ms following start of the perturbation was normalized to the whole stride cycle of the unperturbed stride and analyzed afterwards [34,35] (Fig 2). Additionally, the mean (normalized) EMG-RMS for the right ventral area (VR: RA, EO, IO of right side), left ventral area (VL: RA, EO, IO of left side), right dorsal area (DR: UES, LES, LD of right side) and left dorsal area (DL: UES, LES, LD of left side) was build [23,30]. Reproducibility of the described procedure resulted, exemplarily presented, in an ICC of 0.89 for RMS calculation of the unperturbed stride (muscle group VR). Therefore, the mean EMG-RMS of the three included muscles for each trunk area was calculated. As secondary outcome, co-contraction and coefficient of variation were computed. Co-contraction was analyzed between the ventral and dorsal muscles (formula: mean all ventral muscles / mean all dorsal muscles; V:R) as well as the side right: side left ratio (formula: mean of all right-sided muscles / mean of all left-sided muscles; S_right_:S_left_). The coefficient of variation (CV; EMG-RMS, %; formula: SD (15x EMG-RMS single muscle) / mean (15x EMG-RMS single muscle)) within the 15 perturbations served as the outcome measurement to account for the variability of trunk muscle activity between H and BPP.

In the time domain, the onset of muscular activity (T_ON_; ms) and the time to maximum activity (T_MAX_; ms) were measured, representing a response to the perturbation (Fig 2). A semi-automated detection method (IMAGO process master, LabView®-based, pfitec, biomedical systems, Endingen, Germany) was used to define muscle activity onset [36]. Within this detection method, an increase in the averaged EMG signal (ensemble average; filter: 4^th^ order moving average) of more than 2 standard deviations from baseline level was defined for automatic onset detection. Every automatic detection was controlled through visual inspection. If automatic detection failed (e.g. due to movement artefact), the investigator applied manual correction (<3% of all cases analysed). Besides, onset detection (automatically/manual corrected) was possible during perturbed strides for all twelve muscles in all subjects.

### Kinematic analysis

Segmental trunk motion was measured using a 14-camera 3D-motion analysis system (Vicon, Oxford, UK, MX3, 1000Hz). The kinematic trunk model consisted of 12 markers framing three functional segments (upper thoracic area (UTA), lower thoracic area (LTA) and lumbar area (LA))(Fig 1B)[25]. In addition, four markers framed the pelvis. Marker data were analyzed (Vicon Nexus 1.8) to calculate the relative angles of each segment in relation to the pelvis. The primary outcome measurements were the total motion amplitudes (ROM; [°]) and mean trunk angle (A_mean_; [°]). Both were calculated for normal (unperturbed) as well as perturbed gait for the whole stride cycle and the time interval 200ms following perturbation for extension/flexion (E/F), lateral flexion (LF) and rotation (Ro) of each segment. Reproducibility of the described procedure resulted, exemplarily presented, in an ICC of 0.94 for ROM calculation of the unperturbed stride (LA during rotation) and 0.88 (LA during rotation) for perturbed walking. ROM consisted of the mean of the 15 repetitions following right-sided perturbations. For ROM only, the perturbed step was normalized to the unperturbed step [%] for the whole stride cycle as well as the 200ms after perturbation. The mean trunk angle (A_mean_; [°]) was calculated to describe the overall posture and its 3 segments over the whole stride cycle and 200ms after perturbation. As secondary outcome, the coefficient of variation (CV; ROM, %, formula: SD (15x ROM of each segment per single plane) / mean (15x ROM of each segment per single plane)) within the repeated perturbations was calculated.

**Fig 3:**
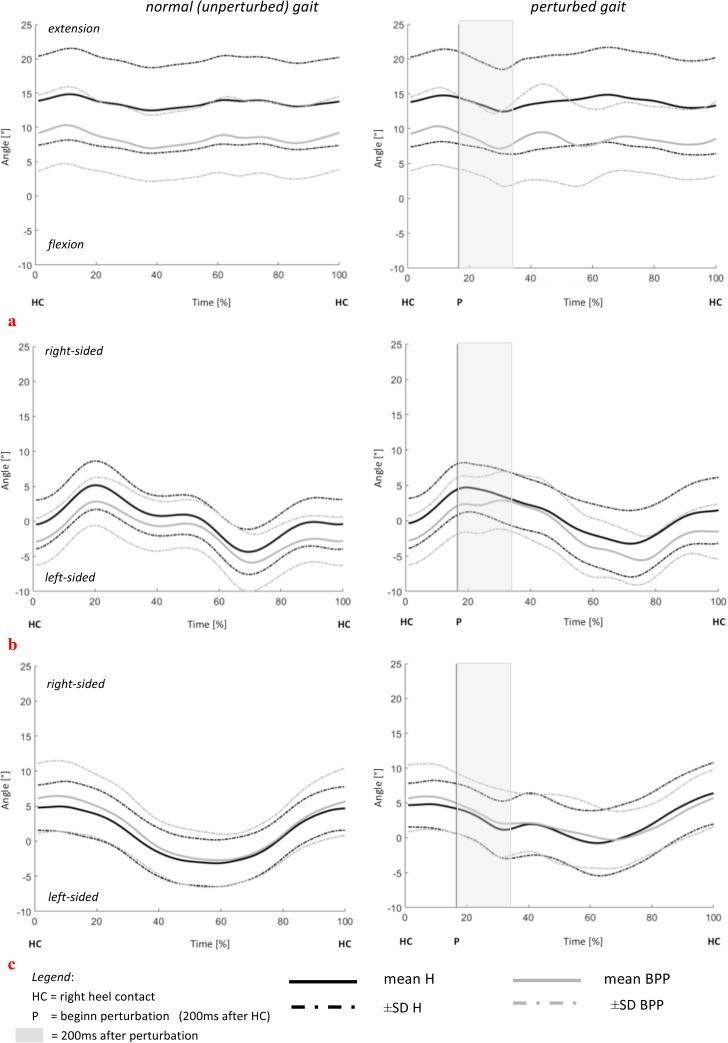
**Group comparison of the LTA segment motion in (a) flexion, (b) lateral flexion and (c) rotation: Comparison of unperturbed and perturbed step in H and BPP (group mean ± SD).** negative values represent flexion, left-sided rotation and left-sided lateral flexion for all 3 segments. positive values represent extension, right-sided rotation and right-sided lateral flexion.

**Fig 4:**
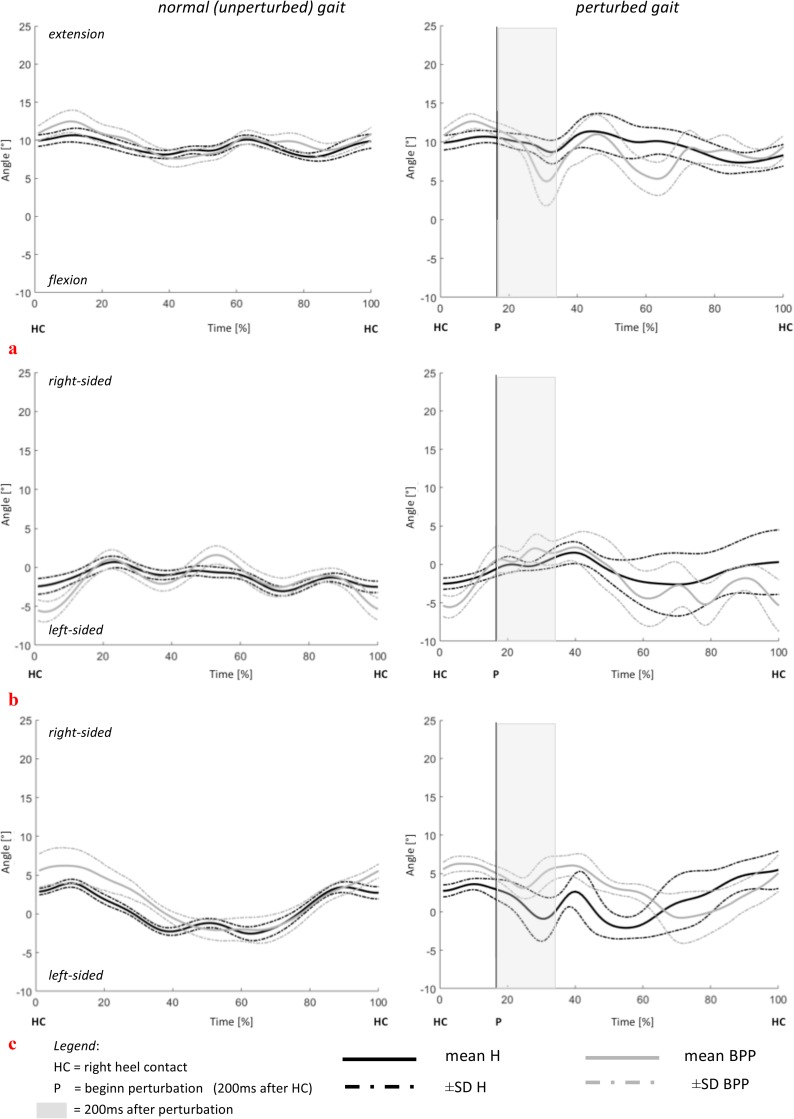
**Case comparison of the LTA segment motion in (a) flexion, (b) lateral flexion and (c) rotation: Comparison of unperturbed and perturbed step in one healthy and back pain patient (individual mean ± SD of 15 perturbed and unperturbed steps).** negative values represent flexion, left-sided rotation and left-sided lateral flexion for all 3 segments. positive values represent extension, right-sided rotation and right-sided lateral flexion.

By showing the full time series of a stride (mean.±SD), the angle-time-curves for the LTA segment (in all planes) during walking and perturbation are presented as a group (H vs. BPP) and single case comparison, to characterize the individuality of the movement pattern in BPP (Figs 3 and 4). With respect to the kinematic model used, negative values represent flexion, left-sided rotation and left-sided lateral flexion for all 3 segments. In contrast, positive values represent extension, right-sided rotation and right-sided lateral flexion.

### Data analysis and statistics

All non-digital data were documented in a paper and pencil-based case report form (CRF) and transferred to the statistical database (JMP Statistical Software Package 9, SAS Institute®). After a plausibility check (range check + extreme value analysis for all outcomes), the data were presented descriptively (means, SD) for all given outcomes. All outcomes were checked for normal distribution with Shapiro-Wilk-Test. The majority of the single muscle / muscle groups (EMG) as well as segment*plane (kinematic) outcomes were normally distributed (e.g. T_ON_ for LD, OE). However, some outcomes were not normally distributed (e.g T_ON_ for IO). Nevertheless, for all outcomes student´s t-test was applied to test for differences between H and BPP (H_matched_ and BPP) based on the knowledge of robustness of the t-test to non-normal distributed data. The level of significance was set at α = 0.05. Due to the use of two primary outcome variables for both, each muscle (EMG: amplitude/latency) or segmental plane (kinematic angle: ROM / A_mean_), multiple testing was controlled via Bonferroni adjustment (adjusted α = 0.025).

## Results

### Back pain

The cohort analyzed represents a back pain prevalence of 15%. Significant differences between H and BPP were present in the pain sub scores (p<0.001) but not in the anthropometrics. Regarding matched group analysis, significant differences between H_matched_ and BPP were present in the pain sub scores (p<0.001), too. In addition, significant differences of acute pain intensity at time of testing (item 1 of pain questionnaire) are present between H (H_matched_) and BPP (p = 0.0001 (H_matched_: p = 0.003)). H (H_matched_) showed an intensity of 0.4 ± 0.8 and BPP of 2.4 ± 2.3 on a numeric rating scale ranging from 0 (no pain) to 10 (maximum pain).

### Trunk muscle activity following stumbling during gait

**Fig 5:**
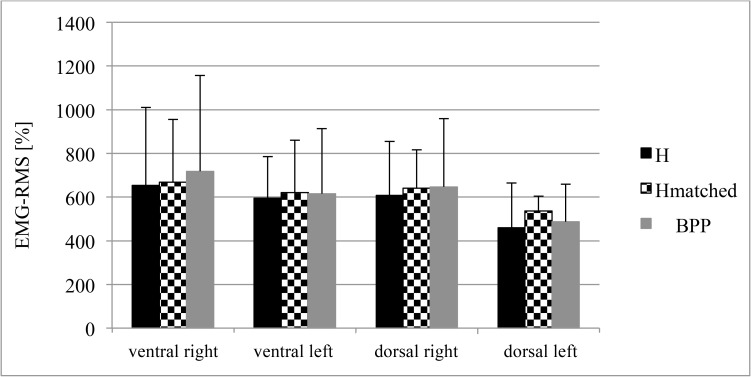
Neuromuscular reflex activity (EMG-RMS; %) of the four trunk areas during stumbling in healthy (H; H_matched_) and back pain symptomatic subjects (BPP). Legend: VR/VL: mean EMG-RMS of RA, EO, IO ri/le; DR/DL: mean EMG-RMS of LD, UES, LES ri/le).

In the EMG-RMS analysis, no group differences (BPP vs. H; BPP vs. H_matched_) regarding the four muscle groups were found (p>0.025)(Fig 5). Co-contraction analysis revealed no differences for V:R and S_rigth_:S_left_ ratio between the groups (p>0.025; V:R ratio: BPP: 1.2 ± 0.6; H: 1.1 ± 0.6; S_right_:S_left_ ratio: BPP: 1.2 ± 0.4, H: 1.2 ± 0.3). Variability of neuromuscular reflex activity, represented by CV, ranged from 23% to 37% for BPP, 25% to 39% for H, and 23% to 39% for H_matched_ without significant differences between groups (p>0.025).

**Fig 6:**
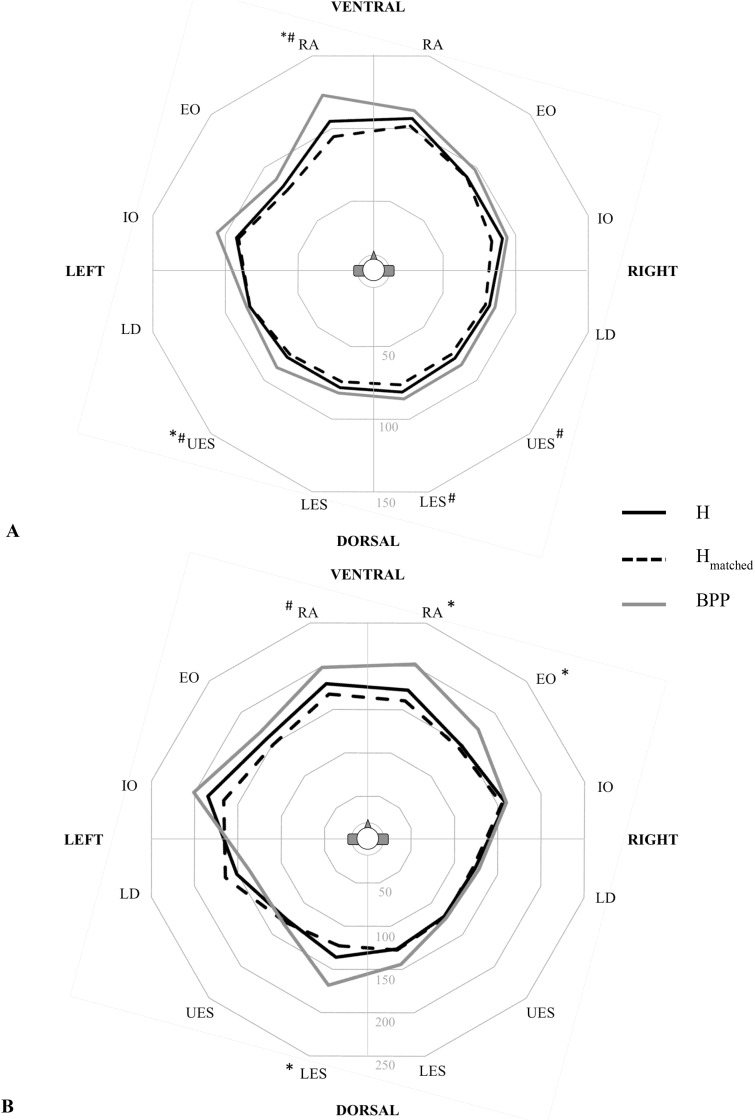
Polarplot of neuromuscular response time of 12 trunk muscles to perturbation. (A) onset of muscle activity (T_ON_; ms) after perturbation. (B) time to maximum activity (T_MAX_; ms) after perturbation.* significant differences between H and BPP (p<0.025); # significant differences between H_matched_ and BPP (p<0.025).

T_ON_ ranged from 78ms to 107ms in H, 74ms to 102ms in H_matched_ and 82ms to 123ms in BPP (Table 2). BPP showed higher latencies for all 12 muscles compared to H, and were significant for RA le (p = 0.019) and UES le (p = 0.005). Matched group analysis of was statistically significant for RA le (p = 0.004), UES le (p = 0.005) and LES ri (p = 0.004)(Table 2; Fig 6A). T_MAX_ showed statistically significant differences between groups (BPP vs. H) for RA ri (p = 0.021), EO ri (p = 0.005) and LES ri (p = 0.016). Matched group analysis (BPP vs. H_matched_) only showed significant differences for RA left (p = 0.004)(Table 2).

**Table 2:** Neuromuscular reflex response (T_ON_; T_MAX_; [ms]) for all muscles for H, H_matched_ and BPP.

outcome measure	group	trunk muscles											
		RA ri	RA le	EO ri	EO le	IO ri	IO le	LD ri	LD le	UES ri	UES le	LES ri	LES le
**T**_**ON**_	**H**	107 ± 18	105 ± 22	91 ± 14	82 ± 12	91 ± 16	93 ± 18	82 ± 9	83 ± 10	79 ± 10	78 ± 10	81 ± 11	78 ± 9
	**H**_**matched**_	102 ± 11	95 ± 13	91 ± 16	79 ± 11	84 ± 12	91 ± 19	79 ± 7	83 ± 10	76 ± 7	76 ± 7	77 ± 9	74 ± 8
	BPP	112 ± 21	123 ± 30	98 ± 15	89 ± 17	94 ± 20	106 ± 31	86 ± 10	85 ± 9	86 ± 9	88 ± 12	86 ± 9	82 ± 12
	p-values BPP vs. H / BPP vs. H_matched_	0.43 / 0.14	0.019 / 0.004	0.09 / 0.24	0.13 / 0.10	0.56 / 0.14	0.04 / 0.16	0.22 / 0.07	0.50 / 0.55	0.046 / 0.005	0.005 / 0.004	0.16 / 0.015	0.24 / 0.07
**T**_**MAX**_	**H**	173 ± 28	180 ± 31	148 ± 22	159 ± 41	159 ± 27	185 ± 37	124 ± 12	151 ± 38	121 ± 17	127 ± 31	126 ± 49	136 ± 91
	**H**_**matched**_	160 ± 28	168 ± 25	144 ± 15	149 ± 27	155 ± 19	166 ± 25	121 ± 13	164 ± 53	122 ± 25	130 ± 35	128 ± 22	123 ± 29
	BPP	203 ± 78	199 ± 23	174 ± 56	169 ± 25	160 ± 31	201 ± 71	128 ± 19	136 ± 23	124 ± 15	133 ± 31	144 ± 17	169± 94
	p-values BPP vs. H / BPP vs. H_matched_	**0.021** / 0.07	0.06 / **0.004**	**0.005** / 0.06	0.42 / 0.72	0.94 / 0.62	0.22 / 0.10	0.29 / 0.30	0.24 / 0.14	0.60 / 0.86	0.58 / 0.84	**0.017** / 0.26	0.27 / 0.10

Outcome measures: T_ON_ = time to onset; T_MAX_ = time to maximum activity

Trunk muscles: Mm rec. abd. (RA), obl. ext. abd. (EO), obl. int. abd (IO) of left and right side; erec. spinae thoracic (T9; UES)/ lumbar (L3; LES), latis. dorsi (LD)

Le = left side; ri = right side

The polar plots (Fig 6) specifies the different muscular reaction patterns of the groups for T_ON_ and T_MAX_.

### Three-dimensional trunk kinematics during normal gait and following stumbling

Trunk motion analysis (ROM) over the whole stride cycle during unperturbed walking revealed no significant differences between groups (p>0.025) (Table 3). However, differences between groups could be found for perturbed step normalized to unperturbed step (whole stride cycle) for the lumbar segment (LA) in lateral flexion with BPP showing higher normalized ROM compared to H_matched_ (p = 0.02; Table 3).

**Table 3:** Total motion amplitude (ROM) during normal, unperturbed step [°] and perturbed step normalized to unperturbed step [%] for the whole stride cycle and the subsequent 200ms after perturbation for all three segments in all planes (mean ± SD).

	Plane	ROM [°] Unperturbed step				ROM [%]							
						Perturbed step normalized to unperturbed step (whole stride cycle)				Perturbed step normalized to unperturbed step (subsequent 200ms after perturbation)			
		BPP	H	H_matched_	p-values BPP vs. H / BPP vs. H_matched_	BPP	H	H_matched_	p-values BPP vs. H / BPP vs. H_matched_	BPP	H	H_matched_	p-values BPP vs. H / BPP vs. H_matched_
**UTA**	**E/F**	9 ± 3	8 ± 3	7 ± 3	0.23 / 0.25	126 ± 10	121 ± 32	127 ± 39	0.63 / 0.95	90 ± 32	89 ± 29	98 ± 39	0.93 / 0.61
	**LF**	11 ± 3	11 ± 3	10 ± 5	0.74 / 0.62	226 ± 99	212 ± 91	174 ± 45	0.63 / 0.13	68 ± 41	65 ± 38	59 ± 29	0.84 / 0.59
	**Ro**	4 ± 2	4 ± 1	5 ± 2	0.83 / 0.86	109 ± 15	123 ± 41	122 ± 48	0.28 / 0.39	117 ± 39	159 ± 80	141 ± 87	0.10 / 0.41
**LTA**	**E/F**	4 ± 1	4 ± 1	5 ± 2	0.95 / 0.44	129 ± 32	118 ± 28	124 ± 11	0.28 / 0.77	90 ± 30	93 ± 37	92 ± 41	0.81 / 0.86
	**LF**	9 ± 2	10 ± 3	10 ± 3	0.35 / 0.50	229 ± 94	209 ± 80	182 ± 14	0.46 / 0,14	239 ± 140	245 ± 194	221 ± 97	0.93 / 0.73
	**Ro**	10 ± 3	10 ± 3	10 ± 3	0.99 / 0.77	99 ± 21	110 ± 28	119 ± 32	0.23 / 0.09	105 ± 24	102 ± 29	101 ± 30	0.81 / 0.73
**LA**	**E/F**	4 ± 1	4 ± 1	4 ± 1	0.65 / 0.37	125 ± 43	123 ± 29	124 ± 18	0.82 / 0.92	154 ± 69	128 ± 64	146 ± 53	0.23 / 0.77
	**LF**	7 ± 3	6 ± 2	6 ± 2	0.23 / 0.23	217 ± 77	183 ± 44	155 ± 34	0.046 **/ 0.02**	265 ± 121	230 ± 90	216 ± 80	0.28 / 0.26
	**Ro**	8 ± 3	8 ± 2	8 ± 2	0.73 / 0.87	113 ± 18	113 ± 20	119 ± 24	0.98 / 0.49	113 ± 37	121 ± 41	131 ± 37	0.52 / 0.27

E/F = extension /flexion; LF = lateral flexion; Ro = rotation.

UTA = upper thoracic area, LTA = lower thoracic area, LA = lumbar area.

negative values represent flexion, left-sided rotation and left-sided lateral flexion for all 3 segments.

positive values represent extension, right-sided rotation and right-sided lateral flexion

The CV for ROM ranged from 39% ± 13% (LA in lateral flexion) to 167 ± 55% (LTA in rotation) for BPP, 44 ± 15% (LA in lateral flexion) to 177 ± 57% (LTA in rotation) for H, and 43 ± 13% (LA in lateral flexion) to 182 ± 60% (UTA in lateral flexion) for H_matched_. No group differences were found (p>0.025).

**Fig 7:**
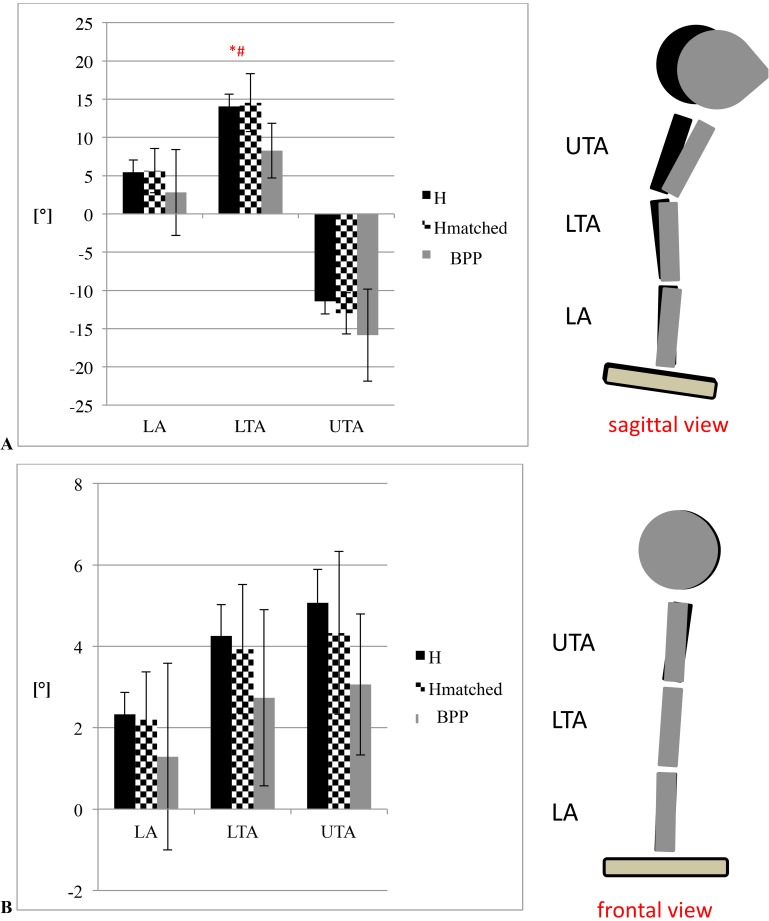
**3-D trunk kinematics during stumbling (mean trunk angle** (A_mean_**; [°]; subsequent 200ms after perturbation) including mean ± 95%-confidence interval for (A) extension-flexion and (B) lateral flexion.** * significant differences between H and BPP (p<0.025); # significant differences between H_matched_ and BPP (p<0.025). negative values represent flexion, left-sided rotation and left-sided lateral flexion for all 3 segments. positive values represent extension, right-sided rotation and right-sided lateral flexion.

Regarding overall posture (A_mean_) during normal gait, BPP showed a significant higher flexed posture of the UTA (p = 0.02) and LTA segment (p = 0.004) during normal walking (Table 4, Fig 3). However, no differences were found for the lumbar segment. Trunk posture analysis (A_mean_) during reflex response showed higher trunk extension (E/F) values in LTA segments for H/H_matched_ compared to BPP (BPP vs H: p = 0.003, BPP vs. H_matched_: p = 0.015; Fig 7A / Table 4).

**Table 4:** Trunk posture (A_mean_ [°]) during normal, unperturbed step and perturbed step for the whole stride cycle and the subsequent 200ms after perturbation for all three segments in all planes (mean ± SD).

Segment	Plane	Unperturbed step				Perturbed step whole stridy cycle				Perturbed step subsequent 200ms after perturbation			
		BPP	H	H_matched_	p-values BPP vs. H / BPP vs. H_matched_	BPP	H	H_matched_	p-values BPP vs. H / BPP vs. H_matched_	BPP	H	H_matched_	p-values BPP vs. H / BPP vs. H_matched_
**UTA**	**E/F**	-17 ± 9	-11 ± 7	-11 ± 6	**0.02** / 0.08	-15 ± 9	-11 ± 7	-13 ± 5	0.08 / 0.42	-16 ± 10	-11 ± 7	-13 ± 5	0.05 / 0.34
	**LF**	-1 ± 2	-1 ± 2	1 ± 2	**0.0006 / 0.02**	-1± 2	2 ± 3	1 ± 3	**0.001** / 0.16	3 ± 2	5 ± 3	4 ± 3	0.05 / 0.31
	**Ro**	2 ± 3	-1 ± 3	-1 ± 3	**0.008** / 0.09	2 ± 4	1 ± 3	1 ± 3	0.30 / 0.51	1 ± 5	-1 ± 3	0 ± 2	0.19 / 0.52
**LTA**	**E/F**	9 ± 7	15 ± 6	15 ± 6	**0.004 / 0.02**	9 ± 6	15 ± 6	15 ± 6	**0.003 / 0.02**	**8 ± 6**	14 ± 6	15 ± 6	**0.003 / 0.015**
	**LF**	-1 ± 3	0 ± 2	0 ± 2	0.13 / 0.38	-1 ± 3	1 ± 3	0 ± 3	0.06 / 0.38	3 ± 3	4 ± 3	4 ± 3	0.12 / 0.33
	**Ro**	2 ± 4	1 ± 2	1 ± 3	0.38 / 0.50	2 ± 4	3 ± 3	2 ± 3	0.71 / 0.84	3 ± 4	3 ± 3	3± 3	0.89 / 0.98
**LA**	**E/F**	3 ± 9	7 ± 6	6 ± 5	0.13 / 0.31	3 ± 9	6 ± 6	5 ± 5	0.24 / 0.39	3 ± 9	5 ± 6	6 ± 5	0.21 / 0.32
	**LF**	-1 ± 3	0 ± 2	0 ± 1	0.22 / 0.50	-1 ± 3	0 ± 2	0 ± 1	0.15 / 0.46	1 ± 4	2 ± 2	2 ± 2	0.18 / 0.44
	**Ro**	1± 4	1 ± 2	1 ± 3	0.80 / 0.76	1 ± 5	2 ± 3	1 ± 3	0.47 / 0.94	0 ± 5	1 ± 3	1± 3	0.64 / 0.75

E/F = extension /flexion; LF = lateral flexion; Ro = rotation.

UTA = upper thoracic area, LTA = lower thoracic area, LA = lumbar area.

negative values represent flexion, left-sided rotation and left-sided lateral flexion for all 3 segments.

positive values represent extension, right-sided rotation and right-sided lateral flexion.

Angle-time-curves, displayed in Figs 3 and 4, visualize the above-described results for the LTA segment in a group (Fig 3) and single case (Fig 4) comparison.

## Discussion

The main purpose of this study was to analyse trunk stability during sudden external loading while walking, characterized by EMG reflex response and three-dimensional segmental motion of the trunk in back pain symptomatic subjects (BPP). This study demonstrates that BPP showed a significantly later onset of some muscle responses to sudden loading without differences in the neuromuscular reflex amplitude. Furthermore, significant differences in patients' overall trunk posture during normal gait and following stumbling were found compared to healthy controls.

A delayed trunk muscle response to sudden force release in BPP is in line with other studies [9,17]. Radebold et al. [16] exemplarily showed an average difference in the response times of approximately 15% (10 ms) between healthy subjects and patients to a sudden, external load applied directly to the trunk in a (half-)seated position. The presented results of the stumbling experiment show comparable differences between H and BPP of 10% to 15%. The controls showed reactions in the area of medium latency with polysynaptic reflex activity (50-80ms) and trigger reactions (80-120ms) [37]. On the other hand, BP patients showed triggered (80-120ms) as well as voluntary reactions (120-180ms) [37]. Besides, the onset with mean values between 74ms and 123ms are systematically higher compared to the quasi-static quick release experiments showing latency of 67ms to 80ms [16]. Nevertheless, this seems appropriate since the perturbation is applied indirectly by the lower extremity. Therefore, it has to be considered that mechanical delay from the distal applied perturbation to the trunk segment might cover these response times. In addition, a prolonged muscle response until maximum activity was found in patients, which has not been previously described elsewhere. This might be discussed as an effect of the delayed onset. However, for a different onset, depending on the activation frequency and recruitment rate, still same time point for the maximum activity can be reached or vice versa. Stumbling while walking represents a functional and daily-life situation in which sudden unexpected loading increases the risk of overloading and injury (e.g. slipping or tripping while walking). The influence of back pain on neuromuscular responses of the trunk to sudden loading could be shown. The majority of studies so far have mostly evaluated static, seated or standing positions, aiming at isolated trunk muscle activity analysis [9,16]. Furthermore, conclusions regarding neuromuscular deficits have mostly been attributed to direct loading of the trunk muscles and have not involved load applied through the extremities such as during gait [9,17,30,34,38]. It might be speculated that the presented results support the use of exercises including indirect loading of the trunk by distal segments ((upper or lower extremities) and not only directly during diagnostics as well as prevention and/or rehabilitation [39].

Detailed neuromuscular response pattern analysis (T_ON_; T_MAX_) proof that patients in particular alter their reaction time of bilateral trunk muscles when the right side is perturbed. Therefore, an impaired neuromuscular control of the trunk has to be assumed. This, in particular, might also have implications for training strategies taking one-sided perturbations into account to address bilateral neuromuscular core muscles while exercising.

No differences in neuromuscular reflex amplitudes could be shown between patients and healthy controls. Lamoth et al. (2005) [40] reported impaired muscle coordination and increased muscle activity of the erector spinae (ES) in low back pain patients compared to healthy participants during normal, unperturbed walking at different velocities. The results of the present study also imply an impaired trunk muscle response to sudden loading in back pain symptomatic subjects while walking but only in the time domain. Additional alterations or restrictions on the magnitude of neuromuscular reflex activity in patients could not be observed. These might be interpreted as a quick-targeted neuromuscular response of healthy participants to sudden walking perturbations. In contrast, delayed BPP response could be caused by pain-inhibited proprioception. Similar EMG amplitudes between groups could stand for an overshooting action to compensate delayed activation onset, despite reduced activation time in the analysed time window. Trunk stability requires using neuromuscular control strategies that include synergistic coactivation and selective recruitment of specific muscles [22]. Therefore, a delayed neuromuscular activity as response to perturbations is associated with reduced stability and therefore a higher risk of developing back pain. Besides, the recently discussed higher variability of muscular activity in patients compared to healthy controls could not be supported by the results presented here [24,40]. Both groups showed high variability in intra-individual neuromuscular reflexes, as calculated by CV over 15 repetitions, in response to the unexpected, high-intensity loading situation [25].

As mentioned above, kinematic outcomes during walking and back pain are diverse [24,26,27,41]. Vogt et al. [27] demonstrated that lumbar trunk motion patterns and displacement in patients while walking were equivalent to those of healthy controls. In contrast, the presented results add, that during normal walking as well as stumbling BP patients show an altered kinematic trunk motion pattern with significant differences, especially for the upper and lower thoracic segment. As shown in this study, these differences are persistent during reflex response to walking perturbations representing a characteristic compensation pattern with counter movements predominantly in sagittal plane. Moreover, significant differences in overall trunk posture (A_mean_) after stumbling in BPP compared to H could be observed for the transversal and sagittal planes. In addition, patients showed a more flexed posture of the trunk, especially in the upper thoracic region. This might be discussed as a relieving or protective posture due to pain or fear of pain involved in walking and repetitive gait perturbations. From a biomechanical perspective, higher flexion angles of the trunk, in relation to the pelvis, imply greater lever arms generating higher moments [42,43]. It could be speculated that this alteration could indicate higher loading during gait perturbations in BPP.

In contrast to others, no differences in the movement variability of inter-segmental trunk motion (ROM) could be demonstrated in BPP [27]. Even though the variability is high in both groups, the absolute ROM is low. Asgari et al. [14] similarly reported no differences in trunk motion variability between low back pain and healthy controls in a flexion-extension task. They demonstrated that a higher movement speed significantly reduced trunk kinematic variability in both groups. With respect to Asgari et al. [14], the high intensity of the chosen perturbation combined with the low overall ROM of the trunk might be responsible for the small differences between healthy controls and back pain symptomatic subjects in the present study [25].

In summary, back pain symptomatic subjects present an altered neuromuscular compensation strategy in response to unexpected sudden loading while walking. In addition, an altered kinematic compensation, especially in the sagittal and transverse planes, is present during normal walking and persistent during sudden loading while walking compared to healthy controls. As a consequence, it might be speculated that the back pain symptomatic subjects were not able to immediately and adequately compensate for repetitive, sudden loading while walking. Hence, BP patients are at potentially higher risk of overloading and injury when exposed to repetitive external loading (e.g. slipping or tripping) [9]. Therefore, it could be speculated, that exercise therapy in prevention and rehabilitation of back pain should include various perturbations with sudden, unexpected loading strategies in participants that are at risk of developing back pain due to unexpected trunk loading [38]. Sensorimotor training (SMT), as described in previous studies including additional external perturbations, seems to be a feasible option for enhancing performance of the trunk muscles [4,44,45]. Further validation of this approach is required by randomized controlled trials.

### Limitations

Certain limitations have to be considered when interpreting the results. During the experiment, all participants walked at the same baseline velocity, not taking into account a potentially reduced self-selected (comfortable) gait velocity in BPP [39]. With respect to standardization, a consistent test situation for all participants was favored anyway [32]. For the data analysis, only right-sided perturbations were analysed. It cannot be ruled out that participants were stressed to different extends due to individual foot dominance. Nevertheless, the human gait is described as an automated and stable movement pattern (high intra-individual reproducibility). Consequently, there is no need to expect asymmetries in participants without pain, complaints and/or injuries at the lower limbs that was ensured by a clinical examination conducted by an experienced physician. Besides, no specific instructions regarding the task of the trunk, legs or arms during compensation were given to the participants. Use of different compensation strategies (e.g. leg-dominant, trunk-dominant) was not assessed. Different strategies might have influenced the presented neuromuscular and kinematic trunk response pattern of both groups to a different amount.

Since we investigated middle-aged active persons, validity of transferring the results to completely untrained or older persons remains unclear. In addition, the calculated BP prevalence of 15% in our sample is based on the categorization by the Korff pain scales and not fully correspondingly to the general use of back pain prevalence. This explains of course the relatively high difference between back pain prevalence in general population and the percentage of subjects categorized to BPP in this study.

Except for the sample size, there were no baseline (anthropometric) differences between groups. The added matched group analysis (BPP vs. H_matched_) did not change the results of trunk EMG and kinematics. Since matching can reduce cofounding factors, it can also eliminate possibly important influencing effects. Therefore, necessity of matching in a cohort of adult participants has to be discussed.

## Conclusion

Back pain symptomatic subjects demonstrate different neuromuscular compensation strategies for sudden loading while walking, presenting increased latencies in muscle reaction without differences in neuromuscular reflex amplitudes. In addition, overall trunk posture, especially in the sagittal and transversal planes, is altered in BPP during normal walking as well as sudden loading. This might be discussed as relieving posture during normal walking and persistent during provoked stumbling. Accordingly, exercise therapy might aim for the improvement of trunk muscle response to sudden unexpected perturbations during dynamic tasks and overall trunk posture during walking. Sensorimotor training in combination with perturbation seems to be suitable. Nevertheless, future research is needed to validate this approach.
